# 

**Published:** 1917-05-26

**Authors:** 


### CONTENTS.

PAGE

**Personal Rights and the Criminal Law Amendment Bill** ... ... ... ... ... ... 141
**The Supply of Doctors** ... ... ... ... ... 142
**Hospital and Institutional News** ... ... ... 143
A National System of Military Hospitals—The Red Cross Service in the Abbey—Manchester Royal Infirmary—Hospital Site Converted into Trenches—The Queen's Hospital for Children and the Lord Mayor—The Employment of Alien Doctors at Hospitals—An Indian on the Staff—The Vitality of the Sunday Funds—The Osteopath Again—Another War Complaint—A Revised Trust Deed for Blackpool Hospital—The Reward of a Courageous Appeal—Workhouse Master as Officer in Charge of a Military Hospital—Hospitals in Munition Areas—New Auxiliary Hospitals in Wales—The Royal Gwent Hospital, Newport—Hampstead General Hospital Secretaryship—Hospital Secretary for the Army—Male V.A.D.s and Military Service—Disabled Fighters' Workshops—The David Little Homestead Memorial—A Memorial to Miss Emily Jackson—A "Children's Bed" at Porth Cottage Hospital—Red Cross Expansion in Devon—Liability to Treat Tuberculosis—Spirit Duty Grant—The Retention of Sufficient Staff—This Week's Drug Market.
**The Anti-Venereal Campaign** ... ... ... 149
The Annual Oration Before the Medical Society.
**Criminality** ... ... ... ... ... ... ... 151
Criminals are Made, not Born.

PAGE

**Soldiers' Heart** ... ... ... ... ... ... ... 152
A Question of Ætiology.
**The Naval M.O. in Action** ... ... ... ... 153
**The Editor's Letter-Box** ... ... ... ... 154
The Irish Anti-College Scheme.
Warrior Patients in Hospitals: their Temperatures and Maladies.
The late Matron and Worcester General Infirmary.
**The Matrons' and Sisters' Department** ... 155
The Preliminary Training School for Nurses: I. Its Uses and Developments.
Round the Hospitals.
Queen Alexandra and Miss Luckes—Misleading British Nurses—Expected College Action—The Irish Anti-College Scheme—Timid Matrons The Nurses of Liverpool Hospitals.
**Liverpool Royal Infirmary**... ... ... ... 157
**Some Problems of To-day**... ... ... ... 158
V. The New Education and its Teachers.
**Addenbrooke's Hospital, Cambridge** ... ... 159
**Parliament and the Profession** ... ... ... 159
**Matrons' and Sisters' Appointments** ... ... 160
**The Book Market** ... ... ... ... ... ... 160
**Ill-health Amongst Plymouth School Children** 160
**The Institutional Worker** ... ... (Suppl.) 1
Answers to March and April Questions.
Enquiries and Answers.

